# Survey of the rate of PSA testing in general practice

**DOI:** 10.1054/bjoc.2001.1962

**Published:** 2001-09

**Authors:** J Melia, S Moss

**Affiliations:** Cancer Screening Evaluation Unit, Institute of Cancer Research, Section of Epidemiology, D Block, Cotswold Road, Sutton, Surrey, SM2 5NG, UK

**Keywords:** prostate cancer, PSA, diagnosis, screening

## Abstract

The use of prostate specific antigen (PSA) test could have a large impact on the incidence of prostate cancer in the UK. Over a period of 1 year (1999), 3.5% out of 160 015 men aged > 45 on a GP database, who had no previous record of prostate cancer, had a PSA test. Of the tested men, 21.3% had a PSA > 4 ng/ml. Future data need to distinguish between men with and without symptoms. © 2001 Cancer Research Campaign http://www.bjcancer.com


*British Journal of Cancer* (2001) **85**(5), 656–657
© 2001 Cancer Research Campaign
doi: 10.1054/ bjoc.2001.1962, available online at http://www.idealibrary.com on http://www.bjcancer.com

### Survey of the rate of PSA testing in general practice

**J Melia and S Moss**

Cancer Screening Evaluation Unit, Institute of Cancer Research, Section of Epidemiology, D Block, Cotswold Road, Sutton, Surrey SM2 5NG, UK

**Summary** The use of prostate specific antigen (PSA) test could have a large impact on the incidence of prostate cancer in the UK. Over a period of 1 year (1999), 3.5% out of 160 015 men aged > 45 on a GP database, who had no previous record of prostate cancer, had a PSA test. Of the tested men, 21.3% had a PSA > 4 ng/ml. Future data need to distinguish between men with and without symptoms. © 2001 Cancer Research Campaign http://www.bjcancer.com

**Keywords**: prostate cancer, PSA, diagnosis, screening

The incidence rate of prostate cancer in the UK has increased over several decades. It is thought that this was partly due to increased detection of early stage cancers through use of transurethral prostatectomy and, in the 1990s, the serum prostate specific antigen (PSA) test to aid diagnosis, mostly in symptomatic men (Chamberlain et al, 1997; Oliver et al, 2000).

The high rate of PSA testing in the USA has been linked to the very high rise in incidence, a shift towards increasing diagnosis of early stage cancers, and more recently a decline in age-specific mortality rates (Collins et al, 2000; Jacobsen et al, 1995), although other factors could have caused the trends in mortality (Oliver et al, 2000). Although the rate of testing is likely to be lower in the UK, there are no routine data with which to assess the extent of PSA testing. Data from a large general practice database (Chamberlain et al, 1997) in the UK, showed that in 1994, 2109 men (1.4%) aged 45 years plus, with no prior history of prostate cancer, had a PSA test noted on their record. More recent data have been examined using the same dataset.

### METHODS

Men aged 45 years plus, with no prior history of prostate cancer or record of a radical prostatectomy, were identified from the UK *MediPlus®* database in 1998 and 1999. The database covered over 150 000 men in this age group registered with around 120 computerized practices. These were a non-random sample of practices spread throughout the UK selected because of their use of the System 5 Torex Health GP computer system. A retrospective analysis was conducted to describe the proportion of males having a PSA test and the levels of PSA recorded. We studied the annual rate of PSA testing using the proportion (%) of all men who had a PSA test within 1 year out of all those in the GP practices.

### RESULTS

Overall, 3.5% of men aged 45 or older who had no previous record of prostate cancer (out of a total sample of 160 015) had a PSA test in a 1-year-period, 1999. The rate increased with age from 1.5% in men aged 45–54 to 5.2% in men aged 70 or older. There was little variation between north and south England (Table 1): the rates overall were 3% and 4%, respectively, although the difference did seem to be greater in men aged 55 or older than in men aged 45–54 years. The numbers of men tested were too small to conduct a detailed analysis by age and region. For all ages, the rate of testing ranged from 0.1% (2 out of 1974) in Oxford to 7.6% (1164 out of 15 417) in Wessex, age standardized to the England and Wales 1997 population aged 45 or older. Among 5626 men who tested for PSA in 1999, the proportion with levels over 4 ng/ml was 21.3% (Table 2), the proportion increasing with age. There was a slight increase in the rate of testing from 2.7% in 1998 to 3.5% in 1999. This increase occurred in all age groups.

### DISCUSSION

The rate of PSA testing in 1999 is more than double the rate reported for 1994 (3.5% compared with 1.4% [Chamberlain et al, 1997]). Limitations with these data include uncertainly about completeness related to non-uniform electronic recording, the possibility that raised PSA results were more likely to be recorded

**Table 1** The proportion of men (%) who had PSA testing over a one year period, 1999, in men with no prior diagnosis of prostate cancer presented by age, and north or south England*

| | | Age (years) | | | |
|---|---|---|---|---|---|
| Area | | 45–54 | 55–69 | 70+ | Total |
| North | % | 1.3% | 3.5% | 4.5% | 3.0% |
| | No. men tested | 269 | 783 | 750 | 1802 |
| | Total in practices | 20 931 | 22 678 | 16 813 | 60 422 |
| South | % | 1.6% | 4.7% | 5.8% | 3.9% |
| | No. men tested | 523 | 1625 | 1488 | 3636 |
| | Total in practices | 31 919 | 34 407 | 25 728 | 92 054 |
| Total | % | 1.5% | 4.2% | 5.3% | 3.6% |
| | No. men tested | 792 | 2408 | 2238 | 5438 |
| | Total in practices | 52 850 | 57 085 | 42 541 | 152 476 |

*North: practices in Mersey, North Western, Northern, Trent, West Midlands and Yorkshire regions; South: practices in East Anglia, North East & West Thames, Oxford, South East & West Thames, South Western and Wessex regions; Scotland and Wales were excluded because of small numbers.

*Received 15 March 2001*
*Revised 10 May 2001*
*Accepted 10 May 2001*

*Correspondence to:* J Melia

**Table 2** The distribution (%) of men in 1999 with a PSA test according to their level of PSA and age

| | Age (years) | | | | | | | |
|---|---|---|---|---|---|---|---|---|
| **PSA test result (ng/ml)** | **45–54 %** | **No.** | **55–69 %** | **No.** | **70+ %** | **No.** | **All %** | **No.** |
| 0–4 | 95.9% | 780 | 84.2% | 2111 | 66.6% | 1534 | 78.7% | 4425 |
| 5–10 | 2.7% | 22 | 10.9% | 274 | 19.3% | 446 | 13.2% | 742 |
| 11–20 | 0.8% | 6 | 2.8% | 71 | 8.1% | 186 | 4.6% | 263 |
| 21+ | 0.6% | 5 | 2.1% | 52 | 6.0% | 139 | 3.5% | 196 |
| Total | 100 | 813 | 100 | 2508 | 100 | 2305 | 100 | 5626 |
| No test recorded | | 54 597 | | 57 545 | | 42 247 | | 154 389 |
| Total in practices | | 55 410 | | 60 053 | | 44 552 | | 160 015 |

than low results, the inability to distinguish between asymptomatic and symptomatic testing, and lack of data on private testing.

The results highlight the lower rate of PSA testing in the UK than in the USA, and this may be associated with the lower registration rate for prostate cancer in the UK. At visits to US primary care physicians in 1995 and 1996, approximately 7% of men aged 60–79 had a PSA test ordered (Collins et al, 2000). In Minnesota, 6.6% of men aged 50 or more in 1992 received their first PSA test, and over 40% of men aged 60 or more had had at least one PSA test in their lifetime (Jacobsen et al, 1995). In the Finnish arm of the European screening trial (Maattanen et al, 1999), 8.5% of men aged 55–67 had a PSA level above 4 ng/ml compared with 15.8% of men aged 55–69 in this survey, suggesting that the latter were more frequently associated with symptoms that might be either related to prostate cancer or more commonly to other conditions such as benign prostatic hypertrophy.

The quality of data available to study the impact of the PSA test for both diagnostic purposes and testing in asymptomatic men on the health service needs to be improved.

At present the use of the PSA test to aid diagnosis may be costing the NHS nearly £2 million annually, but it is unclear what impact this has on the incidence rate and stage of diagnosis. The Department of Health has recently launched an initiative (the PSA Informed Choice Project) to meet the needs for information about the test (http://www.doh.gov.uk/cancer/prostate.htm). It is uncertain what impact this and other initiatives will have on PSA testing in general practice, but data on the rate of PSA testing would help to monitor their effects.

### ACKNOWLEDGEMENTS

We would like to thank Dr Muir Gray for suggesting this study, and Dr Mary Thompson from UK *MediPlus* at IMS HEALTH for help with data extraction. This study was funded by the Department of Health. The views expressed in this publication are those of the authors and not necessarily those of the Department of Health.

### REFERENCES

Chamberlain J, Melia J, Moss S and Brown J (1997) Report prepared for the Health Technology Assessment Panel of the NHS Executive on the diagnosis, management, treatment and costs of prostate cancer in England and Wales. *Br J Urol* **79** (Supp 3): 1–32

Collins MM, Stafford RS and Barry MJ (2000) Age-specific patterns of prostate-specific antigen testing among primary care physician visits. *J Fam Pract* **49**: 169–172

Jacobsen SJ, Katusic SK, Bergstralh EJ, Oesterling JE, Ohrt D, Klee GIG, Chute CG and Lieber MM (1995) Incidence of prostate cancer diagnosis in the eras before and after serum prostate- specific antigen testing. *J Am Med Assoc* **274**: 1445–1449

Maattanen L, Auvinen A, Stenman UH, Rannikko S, Tammela T, Aro J, Juusela H and Hakama M (1999) European randomized study of prostate cancer screening: first year results of the Finnish trial. *Br J Cancer* **79**: 1210–1214

Oliver SE, Gunnell D and Donovan JL (2000) Comparison of trends in prostate-cancer mortality in England and Wales and the USA. *Lancet* **355**: 1788–1789

*© 2001 Cancer Research Campaign*
